# Three-dimensional ultrastructural study of the anther of *Silene latifolia* infected with *Microbotryum lychnidis-dioicae*

**DOI:** 10.1371/journal.pone.0182686

**Published:** 2017-08-09

**Authors:** Hiroki Kawamoto, Aiko Hirata, Shigeyuki Kawano

**Affiliations:** 1 Department of Integrated Biosciences, Graduate School of Frontier Sciences, The University of Tokyo, Kashiwa, Kashiwa, Chiba, Japan; 2 Bioimaging Center, Graduate School of Frontier Sciences, The University of Tokyo, Kashiwa, Kashiwa, Chiba, Japan; Institute for Sustainable Plant Protection, C.N.R., ITALY

## Abstract

When *Microbotryum lychnidis-dioicae* infects a male *Silene latifolia*, *M*. *lychnidis-dioicae* smut spores develop in the pollen sac instead of pollen. In contrast, when *M*. *lychnidis-dioicae* infects a female *S*. *latifolia*, the female flowers become male-like, promoting stamen formation. However, it is unclear when and how *M*. *lychnidis-dioicae* invades the anther. It is important to investigate not only whether hyphae exist when the apical meristem tissue differentiates into flowers and anthers, but also whether hyphae exist when stamen filaments form. We used Grocott’s methenamine silver stain and lectin stain, which stain chitin in the fungal cell wall, to search for *M*. *lychnidis-dioicae* in flower tissues. A few *M*. *lychnidis-dioicae* hyphae were observed intercellularly in the center of the connective of vascular bundles at the early anther developmental stage. Subsequently, large numbers of deeply stained *M*. *lychnidis-dioicae* hyphae were observed intercellularly in the cells surrounding the pollen sac, as well as in the center of the pollen sac. Hyphae stained with lectin were observed intercellularly in all of the stamen filaments at flower development stages. Hyphae were observed in the peduncle connecting the flower and stem. It is thought that *M*. *lychnidis-dioicae* invaded the anther via the stamen filament over a long period. Additionally, in total, 163 sections of connective were obtained, and the cell structure of each anther was colored and subjected to three-dimensional reconstruction. The *M*. *lychnidis-dioicae* hyphae observed in the connective were mainly old hyphae with large vacuoles or dead hyphae (S1 Fig). These hyphae branched out, towards the pollen sac, while growing between the cells. We also observed that the host cells that collapsed near the hyphae had thick cell walls and teliospores. Cell wall collapse and cell degeneration were observed only around hyphae with thick cell walls.

## Introduction

The basidiomycetous genus *Microbotryum* contains many smut fungi members and infects a wide range of dicotyledonous plant hosts belonging to Caryophyllaceae, Dipsacaceae, Lamiaceae, and Lentibulariaceae [1]. *Microbotryum lychnidis-dioicae* is a smut fungus isolated from *Silene latifolia* [2]. *Microbotryum lychnidis-dioicae* has long been used as a model system for the study of ecology and the genetics and propagation of sexual infection [3, 4, 5].

*Silene latifolia* plants infected with *M*. *lychnidis-dioicae* show effects in the floral organs [6, 7, 8]. Different phenotypes appear when *M*. *lychnidis-dioicae* infects female or male *S*. *latifolia* plants. When *M*. *lychnidis-dioicae* infects a male of *S*. *latifolia*, *M*. *lychnidis-dioicae* smut spores are formed instead of pollen in the pollen sac. In contrast, when *M*. *lychnidis-dioicae* infects a female of *S*. *latifolia*, stamen formation is promoted. Subsequently, smut spores are formed instead of pollen in the anthers [6,9].

Smut disease is transmitted by these spores attached to insect carriers [10]. Teliospores, transmitted by insects, germinate after adhering to the flowers or leaves of *S*. *latifolia*. The teliospores form a metabasidium after germination. The metabasidium then forms four basidiospores, which have A1 and A2 sexuality. The basidiospores grow by budding division and the mating of paired basidiospores. Spore germination, meiosis, and the mating of paired basidiospores occur on the host cell surface [11]. Basidiospores form secondary hyphae and invade into the body of the host. After invading the host, *M*. *lychnidis-dioicae* exists in an intercellular form. Subsequently, it is observed in intercellular spaces, vascular bundles, and apical meristematic tissues. The infected *S*. *latifolia* passes the winter with rosette leaves, and *M*. *lychnidis-dioicae* forms teliospores in the anther at flowering time [8, 9, 12].

**Fig 1:**
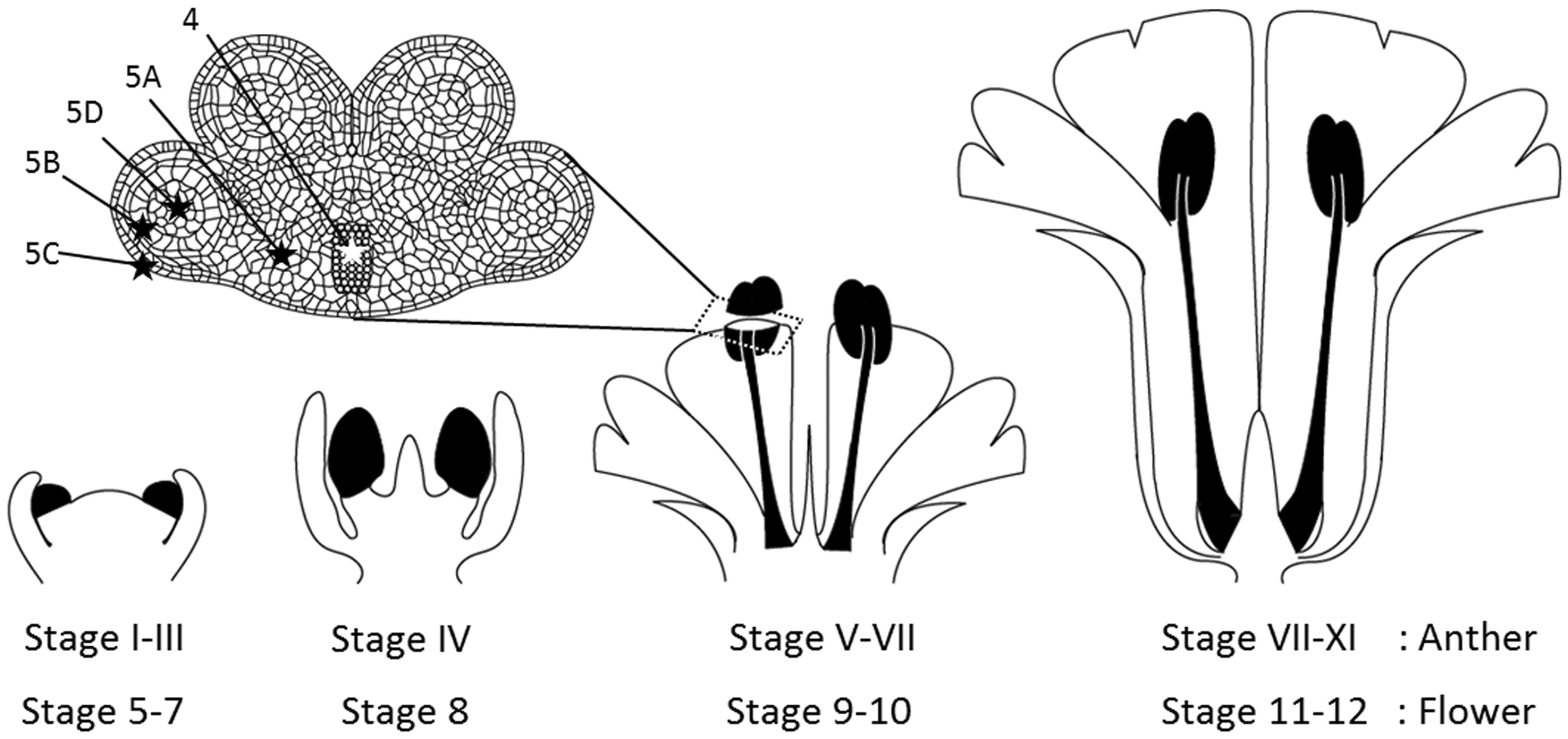
Diagrams of the observation area. The stamen primordia, anther, and filament were observed. Black markings and asterisks indicate the observed areas. 4, 5A, 5B, 5C, and 5D in the anther indicate observation areas in Fig 5. Roman numerals indicate anther developmental stages; Arabic numerals indicate flower developmental stages.

*M*. *lychnidis-dioicae* is observed in apical meristematic tissues in the growing period [12]. In this paper, we used Arabic numerals to indicate the flower developmental stage and Roman numerals to indicate the anther developmental stage (Fig 1). Concurrent with the development of stamens, *M*. *lychnidis-dioicae* hyphae can be observed in the pollen sac in the flower at developmental stage 8 [8]. Dead or dying pollen mother cells are observed and teliospore initiation of *M*. *lychnidis-dioicae* is seen in the same area in the flower at developmental stage 9.

Because it is unclear when *M*. *lychnidis-dioicae* invasion occurs in the anther, it is important to investigate not only whether hyphae exist when the apical meristem tissue differentiates into flowers and anthers, but also whether hyphae exist when the stamen filament and peduncle are formed. In male flowers of *S*. *latifolia* infected with *M*. *lychnidis-dioicae*, tapetal cells disintegrate in the anther at developmental stage VI, unlike normal male flowers, and pollen mother cells collapse before pollen tetrad formation in anther developmental stage VII [7]. In anther developmental stage VIII, the middle layer and the endothelium on the outer side of the tapetum also collapse, and anthers form teliospores instead of pollen. Terminal deoxynucleotidyl transferase dUTP nick end labeling (TUNEL)-positive cell death is observed in tapetal cells. When *M*. *lychnidis-dioicae* makes teliospores in the anther, the tissues constituting the pollen sac collapse, but it is unclear what causes the collapse.

There are several staining methods specific for fungi, such as modified Grocott’s methenamine silver stain, which specifically stains chitin, a component of the fungal cell wall [13, 14] and lectin staining, which uses a fluorescently labeled lectin, wheat germ agglutinin (WGA) [15], but these have not been used before to identify the invasion pathway of *M*. *lychnidis-dioicae*. Grocott’s methenamine silver stain can be used for paraffin wax-embedded samples. WGA is suitable for examining a wide range of tissues. Transmission electron microscopy at high resolution has been used for microstructural observations, such as the localization of *M*. *lychnidis-dioicae* hyphae and the state of the surrounding cells [8]. To observe a specimen with transmission electron microscopy, it is necessary to make a very thin section, so it is not suitable for obtaining three-dimensional (3D) information or for examining hyphae in a range of states. However, it is now possible to acquire ultrastructural images of cell states and fungal localization, and 3D information on fungal hyphae localization using 3D transmission electron microscopy, which is a method of acquiring images of consecutive slices and performing a stereoscopic reconstruction.

In this study, we used modified Grocott’s methenamine silver stain and performed lectin staining, using WGA, to search for *M*. *lychnidis-dioicae* in flower tissues. *Microbotryum lychnidis-dioicae* invaded the anther in anther development stage III, and the hyphae also invaded the anther through the stamen filament. Moreover, we showed that *M*. *lychnidis-dioicae* branched out from connective, adhering to the stamen filament, toward the center of the pollen sac, and host cells collapsed near the hyphae with thick cell walls and teliospores.

## Materials and methods

### Plant materials and plant growth conditions

*S*. *latifolia* seeds were obtained from an inbred line (K-line) and stored in our laboratory. The K-line was propagated for 17 generations of inbreeding to obtain a genetically homogeneous population. Plants were grown from vernalized seeds in pots in a growth chamber at 23°C with a 16-h light/8-h dark cycle.

### *M*. *lychnidis-dioicae* inoculation

Sporidia of A1 and A2 were cultured on potato dextrose agar (BD Difco) at 23°C for 5 days and suspended at 2×10^6^ cells/mL in distilled water. Sporidial mixtures of A1 and A2 at equal concentrations were used throughout the inoculations. Inoculation treatments were carried out on 10-d seedlings of *S*. *latifolia* on 0.8% agar plates. The base of each 10-d old seedling was injected with 2 μL of the mixture. Inoculation was repeated after 3 days. At 3 weeks after inoculation, seedlings were transferred to soil in pots and they were grown in a growth chamber at 23°C with a 16-h light/8-h dark cycle.

### Light microscopy

Whole flower buds were double fixed overnight in 4% glutaraldehyde in 0.1 M phosphate buffer (pH 7.2) at 4°C and post-fixed for 4 h in 2% osmium tetroxide in distilled water. After washing with 0.1 M phosphate buffer (pH 7.2), the fixed flowers were dehydrated in an ethanol series (30, 50, 70, 80, 90, 95, and 100% each step for 15 min at room temperature) and kept in 100% ethanol overnight at 4°C. The ethanol was replaced with xylene and specimens were embedded in paraffin wax. Embedded flowers in paraffin wax were cut into 10-μm sections using a microtome (RV-240, Yamato, Japan). Cutting sections were de-paraffinized in xylene and rehydrated in an ethanol series (100, 95, 90, 80, 70, 50, and 30%; each step for 10 min at room temperature). Rehydrated sections were stained with modified Grocott’s methenamine silver stain in accordance with Pintozzi et al. [14]. WGA lectin staining was performed in native plant tissue, and the plant tissue crushed in the slide glass in accordance with Meyberg et al. [15]. Decolorization was not performed prior to WGA staining. Because the chloroplasts are underdeveloped in the observation area, observation is possible without bleaching treatment. The sections and crushed tissue on the slide glass were observed with a microscope (BX60, Olympus, Tokyo, Japan).

### Transmission electron microscopy

Flower buds were dissected with fine forceps, and only the anthers were removed, placed in 1-hexadecene, and frozen in a high-pressure freezing machine (HPM010, BAL-TEC) that was cooled with liquid nitrogen (-196°C). The samples were immediately transferred to 2% osmium tetroxide in dry acetone at 0°C and incubated at -80°C for 100 h. The samples were then gradually warmed from -80°C to 0°C over 5 h, held for 1 h at 0°C, warmed again from 0°C to 23°C over 1 h, and incubated for 1 h at 23°C (Leica EM AFS, Leica Microsystems). In addition to the high-pressure freeze-fixation method, we performed chemical fixation. Anthers were fixed in 2.5% glutaraldehyde for 2 h at room temperature (rt). After the anthers were washed four times with 0.1 M sodium cacodylate buffer (pH 7.2), the anthers were post-fixed with 1% osmium tetroxide for 2 h at room temperature. The samples were washed three times with dry acetone at room temperature, infiltrated with increasing concentrations of Spurr’s resin in dry acetone, and finally infiltrated with Suppr’s resin. Ultra-thin sections (100 nm) were cut with a diamond knife (DIATOME Ltd.) and mounted onto Formvar-coated copper grids. The sections were stained with 3% uranyl acetate for 2 h at room temperature and examined using an electron microscope (H-7650, Hitachi, Tokyo, Japan) at 100 kV. Each subcellular component in the digital TEM images was traced manually using Procreate for iPad (Savage Interactive Pty. Ltd., Tasmania, Australia). 3D images were subsequently reconstructed using the TRI/3D SRF III software (Ratoc System Engineering, Co., Ltd., Tokyo, Japan).

## Results

### Determining the invasion time of *M*. *lychnidis-dioicae* in anthers

**Fig 2:**
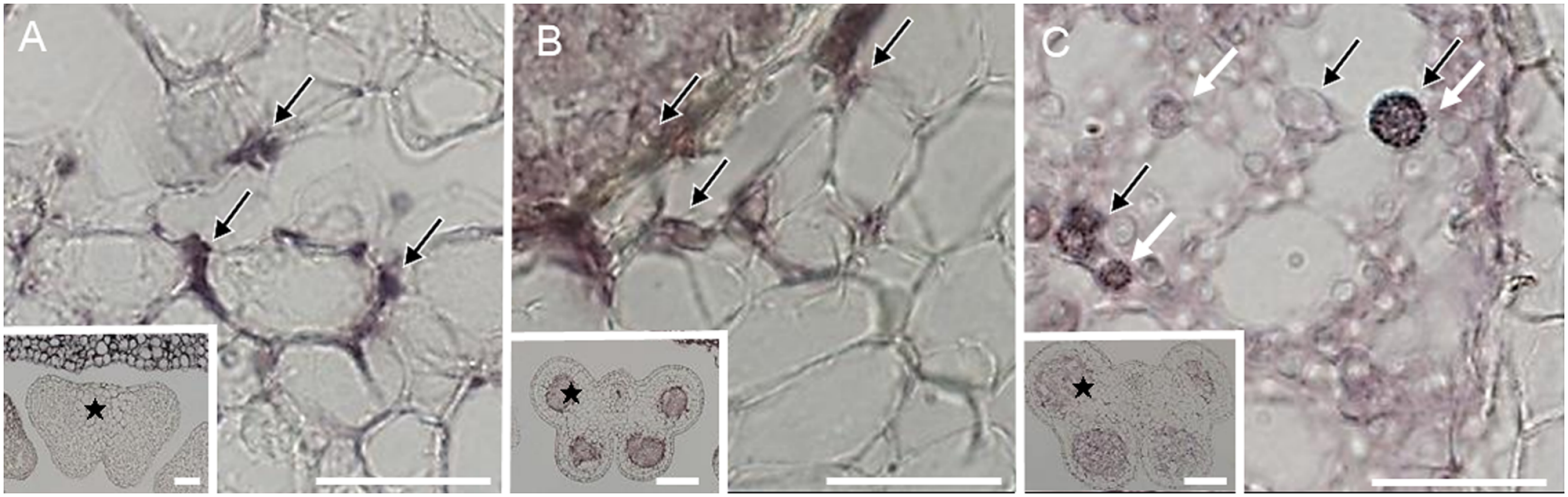
Modified Grocott’s methenamine silver-stained anther tissues from an infected male, showing the purple hyphae of *Microbotryum violaceum* invading the male flower anthers of *Silene latifolia*. Fungal hyphae and spores stained by modified Grocott’s method were detected in the intercellular region and pollen sacs. Stars in inset indicate the observed area. Black arrows indicate smut fungus. White arrows indicate teliospore. (A) connective in stage III, (B) pollen sac in stage VII, (C) pollen sac in stage IX. Bars = 50 μm.

In this paper, we used Arabic numerals to indicate the flower developmental stage and Roman numerals to indicate the anther developmental stage. Stamen filaments are formed in flower developmental stage 8 and subsequently extend in flower developmental stage 9. In the stamens, the stamen primordia are formed in developmental stage 5, and four pollen sacs are formed in flower developmental stage 9. Flower developmental stages 5 to 9 correspond to anther developmental stages I to V. Four layers of the epidermis, the endothelium, the middle layer, and the tapetum are formed so as to enclose the pollen mother cells in the pollen sac (Fig 1). The four pollen sacs are connected by connective (Fig 1). The connective is connected to the stamen filament in the central part of the anther. Vascular bundles exist in the central part of the connective. The connective, which connects stamen filaments, is a key aspect of the infection pathway of *M*. *lychnidis-dioicae*. We observed *M*. *lychnidis-dioicae* localization on paraffin wax-embedded tissue sections in anther developmental stages III, VII, and IX using modified Grocott’s methenamine silver stain (Figs 1 and 2).

It is possible to stain chitin, which is a component of the fungal cell wall, on a paraffin wax-embedded samples using modified Grocott’s methenamine silver stain. We observed a small number of *M*. *lychnidis-dioicae* hyphae in the center of the connective, which had vascular bundles in anther developmental stage III (Fig 2A). Many *M*. *lychnidis-dioicae* hyphae, which were deeply stained, were observed intercellularly among the cells surrounding the pollen sac and in the center of the pollen sac in anther developmental stage VII (Fig 2B). A spore of *M*. *lychnidis-dioicae*, which has the specific shape of a teliospore, was observed in the center of the pollen sac in anther developmental stage IX (Fig 2C), but was not observed in anther developmental stage VII (Fig 2B and 2C). Thus, these findings suggest that the hyphae of *M*. *lychnidis-dioicae* migrate from the connective to each pollen sac and then form teliospores in the pollen sac.

### Penetration of *M*. *lychnidis-dioicae* into the anther during later development

**Fig 3:**
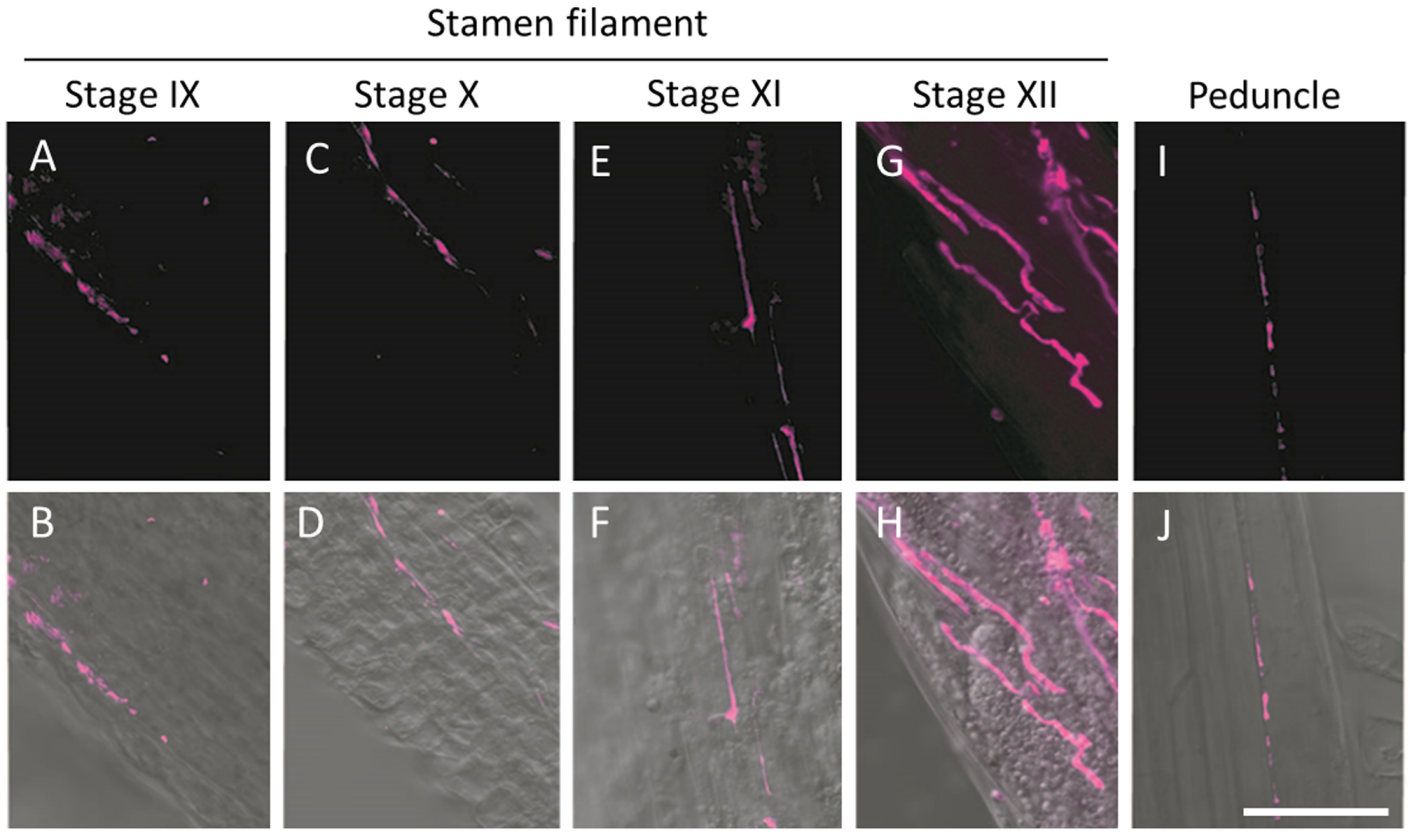
Hyphae localization in the stamen filament of plant developmental stages 9–12 and the peduncle. Fungal hyphae were observed in the stamen filament at stages 9–12. Fungal hyphae were also observed in the peduncle at stage 12. (A, C, E, G, I) Stained hyphae using WGA labeled with Alexa 647; (B, D, F, H, J) Merged images.

Hyphae of *M*. *lychnidis-dioicae* grow rapidly in the anther at anther development stage VII and later (Fig 2B) [8]. The hyphae of *M*. *lychnidis-dioicae* probably invade the anther later, even after the stamens have differentiated. To clarify whether penetration into the anther occurred after stamen formation, we investigated *M*. *lychnidis-dioicae* localization in the stamen filament during flower developmental stages 9–12 (Figs 1 and 3). The stamen filament extends during these stages.

Because modified Grocott’s methenamine silver stain requires tissue sectioning, it is difficult to search for hyphae over a wide range in a thin cylindrical structure like the stamen filament. Thus, we performed staining using WGA lectin, which can stain hyphae in native plant tissue, and then crushed the tissue to search for hyphae of *M*. *lychnidis-dioicae* in the stamen filament and peduncle. Because stamens have no chloroplasts, we used WGA labeled with Alexa 647 to detect fluorescence in the infrared range, thereby reducing auto-fluorescence and allowing accurate detection of hyphae. In all stamen filaments in flower developmental stages 9–12, hyphae stained with the lectin were observed intercellularly in the stamen filament (Fig 3A–3H). Additionally, the number of hyphae in stamen filaments increased from stages 9 to 12. Moreover, hyphae were observed even in the peduncle, connecting flowers and stems (Fig 3I and 3J). Thus, it seemed clear that *M*. *lychnidis-dioicae* invaded the anther through the stamen filament and peduncle even after the stamens were formed.

### Detailed observation of hyphae in connective: A 3D ultrastructural study

**Fig 4:**
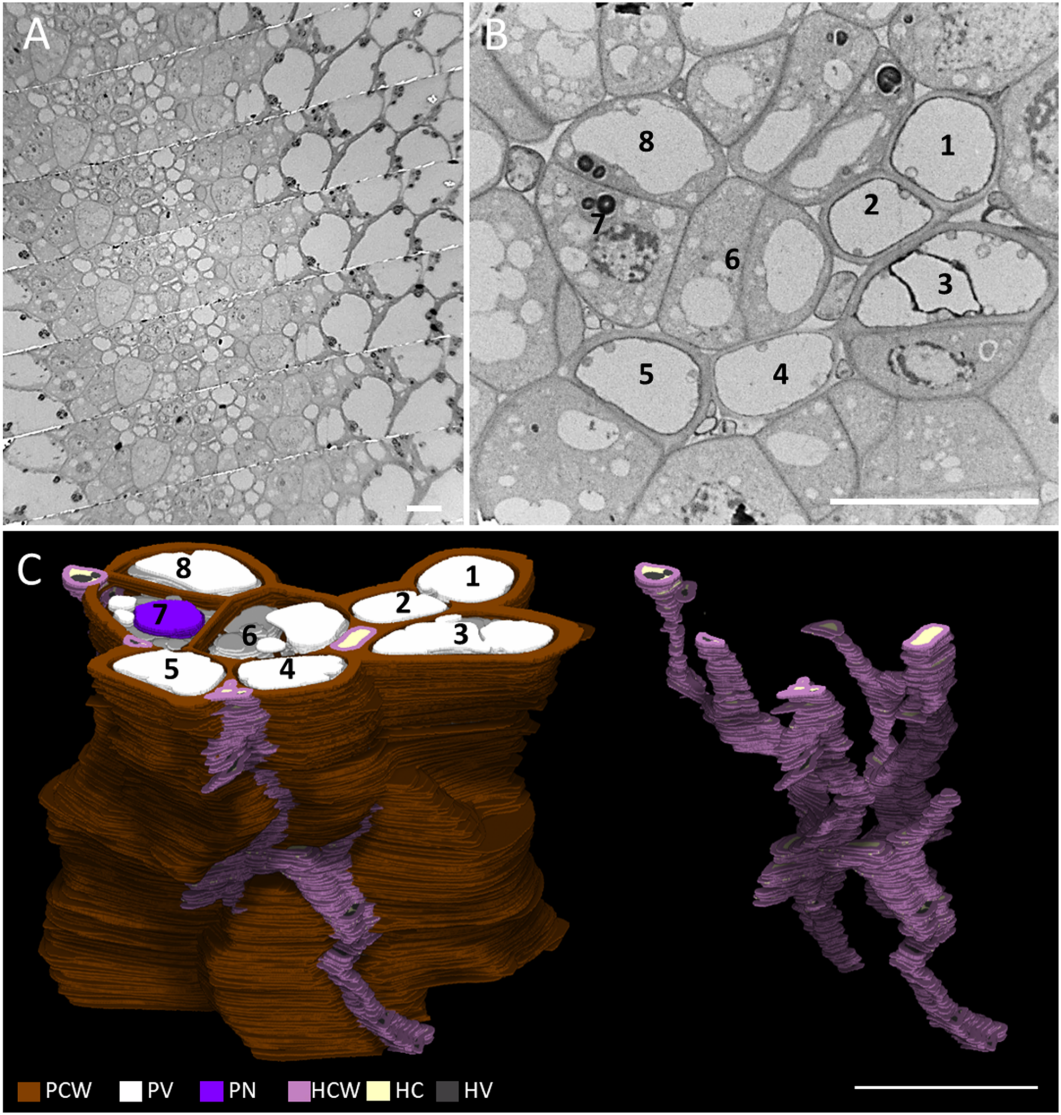
Three-dimensional ultrastructural study of connective in an infected male *S*. *latifolia*. We observed connective using a serial-section method. (A) Low-magnification TEM images, (B) high-magnification of TEM images, (C) 3D reconstructed images. Numbers indicate the same position in b and c. The cells, indicated by numbers, are sieve and companion cells. PCW, plant cell wall; PV, plant vacuole; PN, plant nucleus; HCW, hyphae cell wall; HC, hyphae cytoplasm; HV, hyphae vacuole. Bars = 10 μm.

We acquired serial sections of connective, a key point of infection of *M*. *lychnidis-dioicae*, using transmission electron microscopy. In total, we obtained 163 cross-sections in the connective in stage VII (Fig 4A). The cell structure of each anther was colored and a 3D reconstruction was performed (Fig 4B and 4C). In the images, brown color indicates plant cell wall, white indicates vacuoles, indigo indicates plant nucleus, plastids are blue, violet indicates hyphae, light yellow indicates hyphal cytoplasm, hyphal vacuoles are gray, and light blue indicates the hyphal nucleus. In the connective, we observed sieve cells and companion cells that are part of the phloem tissue in the anthers at anther developmental stage VII. Hyphae of *M*. *lychnidis-dioicae*, shown in purple, were seen intercellularly in connective (Figs 2A, 4C and S1 Movie). The *M*. *lychnidis-dioicae* hyphae in the connective were primarily old hyphae with large vacuoles and dead fungal cells 5 (S1 Fig). These hyphae were branching out toward the pollen sac, between the cells (Fig 4C and S2 Movie).

### Morphology of *M*. *lychnidis-dioicae* in the pollen sac and its influence on surrounding cells

**Fig 5:**
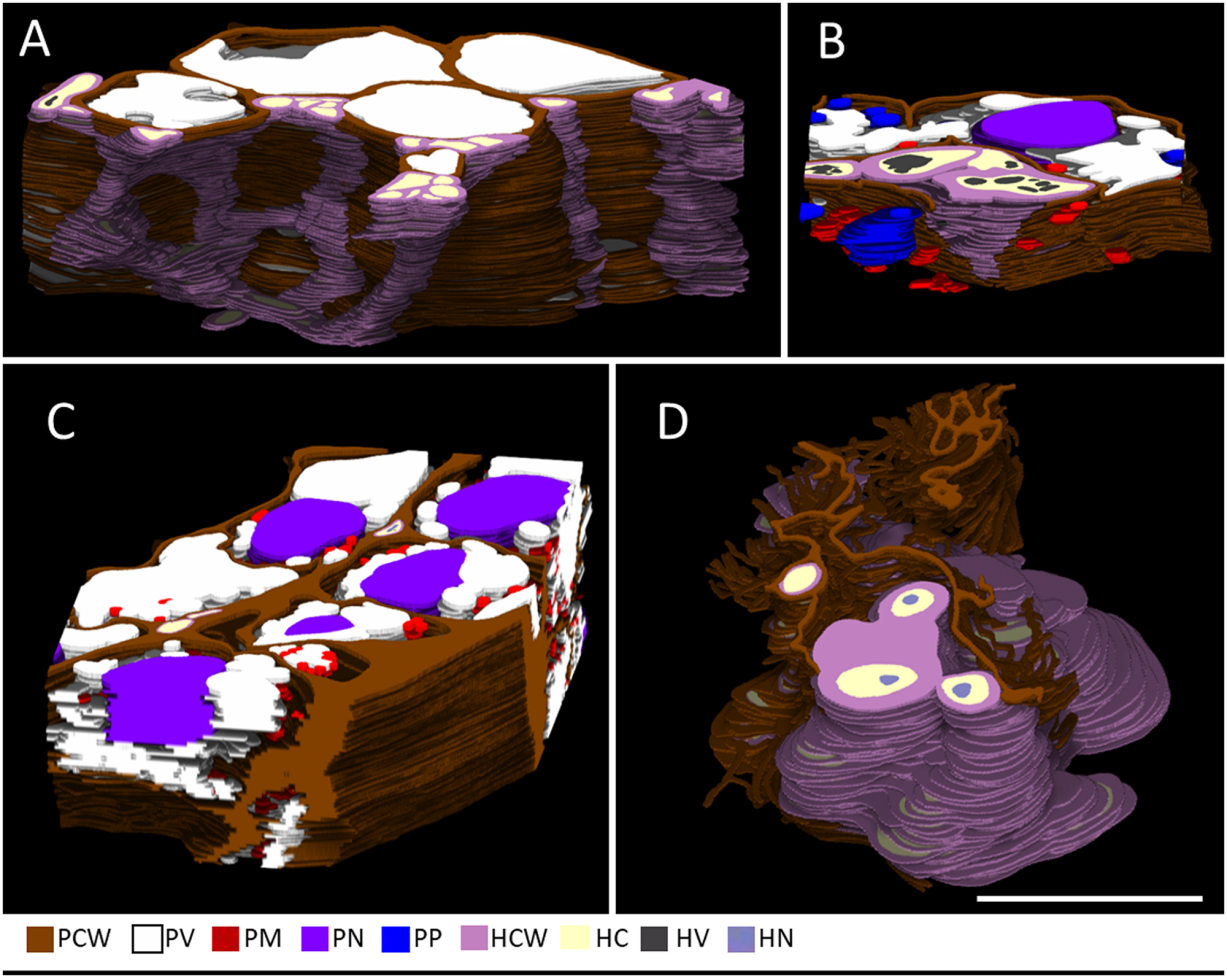
3D ultrastructural study of pollen sacs in infected male in stages VI-VIII. We observed pollen sacs in an infected male using a serial-section method. PCW indicates plant cell wall. (A) 3D reconstructed image of the region between the endothelium and connective, (B) 3D reconstructed images of the region between the tapetum and the middle layer, (C) 3D reconstructed images of the region between the epidermis and endothelium, (D) 3D reconstructed images of the region in the center of the pollen sac. PV, plant vacuole; PM, plant mitochondrion; PN, plant nucleus; PP, plant plastid; HCW, hyphae cell wall; HC, hyphae cytoplasm; HV, hyphae vacuole; HN, hyphae nucleus. Bar = 10 μm.

We obtained 92 sections between the endothelium and connective in anther developmental stage VII (Fig 5A). Unlike the vascular bundles of connective, *M*. *lychnidis-dioicae* hyphae had small vacuoles in the interior of the connective and the endothelium, located on the way from the connective toward the pollen sac in anther developmental stage VIII (Fig 5A, S3 Movie and S2 Fig). These hyphae were considered to be relatively new. The hyphae were elongated, while branching out toward the pollen sac.

**Fig 6:**
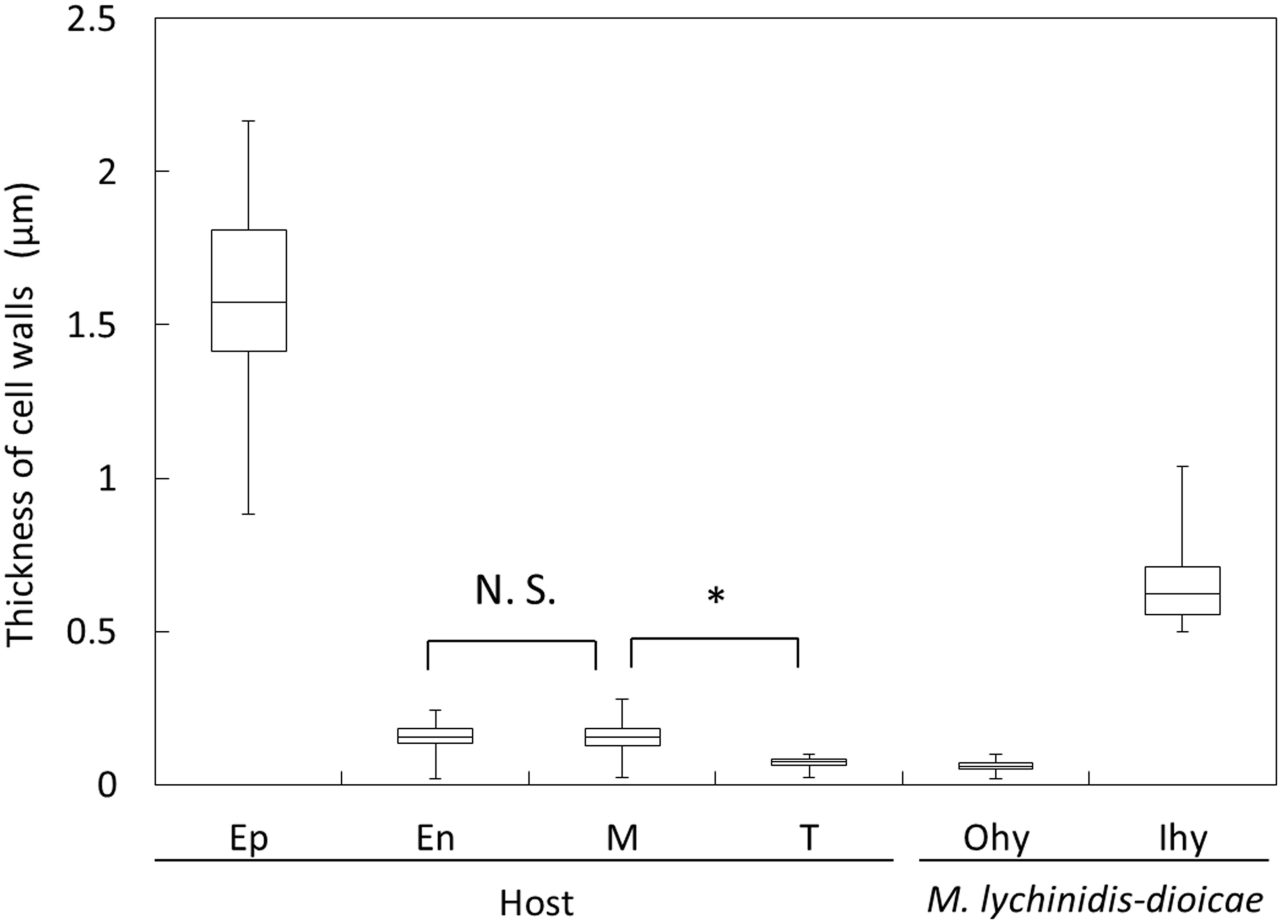
Thickness of cell walls in host cells, and inner and outer localized *M*. *lychnidis-dioicae*. We measured the thickness of cell walls of *S*. *latifolia* cells and inner and outer hyphae of *M*. *lychnidis-dioicae* in the anther. We measure 50 individuals and sections at each organization. Ep, epidermis; En, endothelium; M, middle layer; T, tapetum; Ohy, outer hyphae; Ihy, inner hyphae. N.S.: not significant, * p < 0.01 (by The Mann-Whitney U-test)

We acquired 28 sections between the tapetum and the middle layer in the anther developmental stages VI to VII (Fig 5B). Hyphae with thick cell walls were observed between the tapetum and the middle layer from stages VI to VII. The thickness of the cell wall of 50 individuals at each organization in the electron microscopic images was measured using the ImageJ software (Fig 6). We found that the cell walls of hyphae in the pollen sac were thicker than those of hyphae in connective. The cell walls of tapetal cells collapsed near the hyphae with thick cell walls (Fig 5B, S4 Movie and S2 Fig).

We acquired 44 sections between the epidermis and the endothelium in anther developmental stages VI to VII (Fig 5C). In anther developmental stages VI to VII, hyphae with thin cell walls were observed between the endothelium and the epidermis at the same time as the disintegration of tapetal cells (Fig 5C, S5 Movie and S2 Fig). However, the endothelium and the epidermis were healthy in anther developmental stages VI to VII (Fig 5C, S5 Movie and S2 Fig). Hyphae with thick cell walls were observed between the endothelium and the epidermis in anther developmental stage XII. The pollen sac was occupied with teliospores at this stage. The cell walls of the epidermis and the endothelium did not disintegrate near the hyphae with thick cell walls, but organelles in the cells disappeared, and aggregation of nuclei and degeneration of whole cells were observed in anther developmental stage XII (S3 Fig and S6 Movie).

We acquired 44 sections in the center of the pollen sac, where the pollen mother cells were located in anther developmental stage VII (Fig 5D). Tapetum and pollen mother cells had completely disintegrated after anther developmental stage VII. Hyphae with thick cell walls existed in the center of the pollen sac, where the pollen mother cells were located. Additionally, collapsed cells and cell wall remnants were observed in the center of the pollen sac (Fig 5D, S7 Movie and S2 Fig).

## Discussion

### Timing of *M*. *lychnidis-dioicae* invasion of the anther

To clarify when and how *M*. *lychnidis-dioicae* invades the pollen sac, we comprehensively searched the hyphae with an optical microscope after specifically staining the hyphae. We used modified Grocott’s methenamine silver stain and lectin staining using WGA. Modified Grocott’s stain and WGA highlight fungal cell walls [14] [15]. We concluded that hyphae invade from the connective into the pollen sac after the pollen sac is formed, because hyphae of *M*. *lychnidis-dioicae* were confirmed only in connective, not in the pollen sac locule in anther developmental stage III (Fig 2). The hyphae of *M*. *lychnidis-dioicae* differed from those observed in anther developmental stages VII and IX (Fig 2). The amount of chitin, which covers cell walls, was relatively reduced, so that it was lightly stained with modified Grocott’s methenamine silver stain because teliospore spores, composed of hyaline matrix, were being formed in anther developmental stage IX [13]. Additionally, *M*. *lychnidis-dioicae* hyphae were observed in the stamen filament in flower developmental stages 9 to 12 and in the peduncle in flower developmental stage 12, after the stamens were formed (Fig 3). *Microbotryum lychnidis-dioicae* formed teliospores after flower developmental stage 9 [8]. At that time, tapetal cells and pollen mother cells collapse rapidly, and *M*. *lychnidis-dioicae* hyphae grow (Fig 3B and 3C) [8]. It is thought that *M*. *lychnidis-dioicae* hyphae invade the anther after the formation of the anther, as observed in this study, rather than that the hyphae grow rapidly. Hyphae with thin cell walls were observed in the vascular bundle of connective, adhering to the stamen filament (Fig 4). Both hyphae with large vacuoles and hyphae with few vacuoles were present in the connective. Hyphae with large vacuoles are older, and those with few vacuoles are newer; hyphae with large vacuoles were present in the connective (Fig 4C, 4D and S1 Fig), but the hyphae around the pollen sac had fewer vacuoles (Fig 5A and S1 Fig). Thus, we suggest that *M*. *lychnidis-dioicae* invaded the anther after flower developmental stage 9.

### Two types of morphological changes in cells in the pollen sac

Using electron microscopy, we found *M*. *lychnidis-dioicae* hyphae at the center of the pollen sac at flower developmental stage 8, and found teliospores of *M*. *lychnidis-dioicae* at the center of the pollen sac at flower developmental stage 9 [9]. Flower developmental stages 8 to 9 correspond approximately to anther developmental stages IV to VI. Tapetal cells collapsed at anther developmental stage VI, which is faster than in a healthy male, and the pollen mother cells also collapse before pollen tetrad formation in anther developmental stage VII in male flowers [7]. After anther developmental stage VIII, the middle layer and the endothelium were on the outer side of the tapetum, and *M*. *lychnidis-dioicae* formed a teliospore instead of pollen. In our previous study, TUNEL-positive cell death was detected in the tapetal cells [7]. However, necrosis-like TUNEL-negative cell death occurred in pollen mother cells, the middle layer, and the endothelium that disintegrated with *M*. *lychnidis-dioicae* infection [7]. In our 3D ultrastructural study, the collapse and degeneration of cells were confirmed in pollen mother cells, the tapetum, the middle layer, and the endothelium in anthers infected with *M*. *lychnidis-dioicae* (Fig 5B and S2 Fig). The cell wall collapses in the tapetum in anther developmental stage VI (Fig 5B). The pollen mother cells died and cell wall remnants were observed in anther developmental stage VII (Fig 5B and 5D). The middle layer and the endothelium remained healthy in anther developmental stage VI. However, the cell walls did not disintegrate, but the cells were degenerate, in the endothelium in anther developmental stage XII (S3 Fig).

Based on measuring the thickness of the cell wall of the host, the average of the thickness of the middle layer was 154.5 nm (Fig 6). Moreover, the average of the thickness of the tapetal cells was 73 nm (Fig 6). The cell wall of the secretory tapetal cells in *Arabidopsis thaliana* decreases before meiosis, and the cell walls of pollen mother cells also decrease at the same time [16]. Meiotic division also occurs in *S*. *latifolia* at anther developmental stage VI. It is thought that the cell walls collapse because the cell wall of the tapetal cell is quite thin in anther developmental stage VI (Fig 5B) [7]. However, in the endothelium, the cell walls did not collapse when the cells had degenerated because the cell walls and the intermediate layer were thicker than those of the tapetum.

### Collapse of cell walls in pollen mother cells and tapetal cells

Teliospores arise from secondary hyphae [13]. When a teliospore is formed, it is covered with hyaline matrix, called a sheath [13]. It is thought that teliospores are involved in the collapse of pollen mother cells because pollen mother cells disintegrate at the same time as the appearance of the teliospore [8]. In this 3D ultrastructural study, when *M*. *lychnidis-dioicae* hyphae were observed in the central part of the pollen sac, cell collapse was also observed (Fig 5). The average of the thickness of the cell wall of the hyphae, observed around the collapsing cells, was 624 nm (Fig 6). Only hyphae with thick cell walls were observed near the cell wall remnants of the pollen mother cells and tapetal cells in the pollen sac (Fig 5B and 5D). Corn smut, *Ustilago maize*, invades into the interior of host cells [6]; however, we confirmed that *M*. *lychnidis-dioicae* was only present intercellularly; it did not penetrate into the interior of host cells.

The average of the thickness of the cell walls of *M*. *lychnidis-dioicae* hyphae was 60 nm between the epidermis and the endothelium and connective in anther developmental stage VI (Fig 6). Where hyphae with thin cell walls were present, the cells had not disintegrated (Fig 5D). However, cell degeneration was observed near hyphae with thick cell walls, with teliospore formation, between the epidermis and endothelium, in anther developmental stage XII (S2 Fig). Hyphae of *M*. *lychnidis-dioicae* have two kinds of vacuoles when forming teliospores [8]. Thus, it is thought that the teliospore sheath or the teliospore formation process is related to the collapse of host cells and the degeneration of cells. These results support the hypothesis that the presence of the teliospore induces the collapse of cell walls and the degeneration of cells because these features were observed only around hyphae with thick cell walls.

## Supporting information

S1 Fig Morphological differences in fungal cells between the center of the connective and the connective near the pollen sac. The center of the connective contains more dead fungal cells than does the connective near the pollen sac. Black arrows indicate old hyphae with large vacuoles and dead hyphae. (A) Center of the connective, (B) Connective near the pollen sac. Bars = 5 nm.
(TIF)

S2 Fig TEM images in a three-dimensional ultrastructural study of pollen sacs in infected males in stage VI-VIII. We observed pollen sacs in infected males using serial sections. (A) TEM images using three-dimensional reconstructed images between the endothelium and connective, (B) TEM images using three-dimensional reconstructed images between the tapetum and the middle layer, (C) TEM images using three-dimensional reconstructed images between the epidermis and endothelium, (D) TEM images using three-dimensional reconstructed images in the center of the pollen sac. Bar = 5 μm.
(TIF)

S3 Fig Three-dimensional ultrastructural study of pollen sacs in infected male in stage XII. We observed between the epidermis and endothelium in infected male using the serial-section method. PCW: plant cell wall, PV: plant vacuolar, PN: plant nucleus, HCW: hyphae cell wall, HC: hyphae cytoplasm, HV: hyphae vacuolar. Bar = 10 μm.
(TIF)

S1 Movie 3D reconstruction movie of the connective tissues. Color legends used in the movie are the same as in Fig 4.
(AVI)

S2 Movie 3D reconstruction movie of *M*. *lychinidis-dioidae* in the connective tissues. Color legends used in the movie are the same as in Fig 4.
(AVI)

S3 Movie 3D reconstruction movie between endothelium and connective tissues. Color legends used in the movie are the same as in Fig 5A.
(AVI)

S4 Movie 3D reconstruction movie between tapetum and middle layer. Color legends used in the movie are the same as in Fig 5B.
(AVI)

S5 Movie 3D reconstruction movie between epidermis and endothelium. Color legends used in the movie are the same as in Fig 5C.
(AVI)

S6 Movie 3D reconstruction movie between epidermis and endothelium at the pollen sac in infected male in anther developmental stage XII. Color legends used in the movie are the same as in S3 Fig.
(AVI)

S7 Movie 3D reconstruction movie of the center of the pollen sac. Color legends used in the movie are the same as in Fig 5D.
(AVI)
