# Supplementary figures and images for: Three-dimensional ultrastructural study of the anther of Silene latifolia infected with Microbotryum lychnidis-dioicae
Source: PLoS One. 2017 Aug 9;12(8):e0182686. doi: 10.1371/journal.pone.0182686 (PMC5549918; doi:10.1371/journal.pone.0182686)

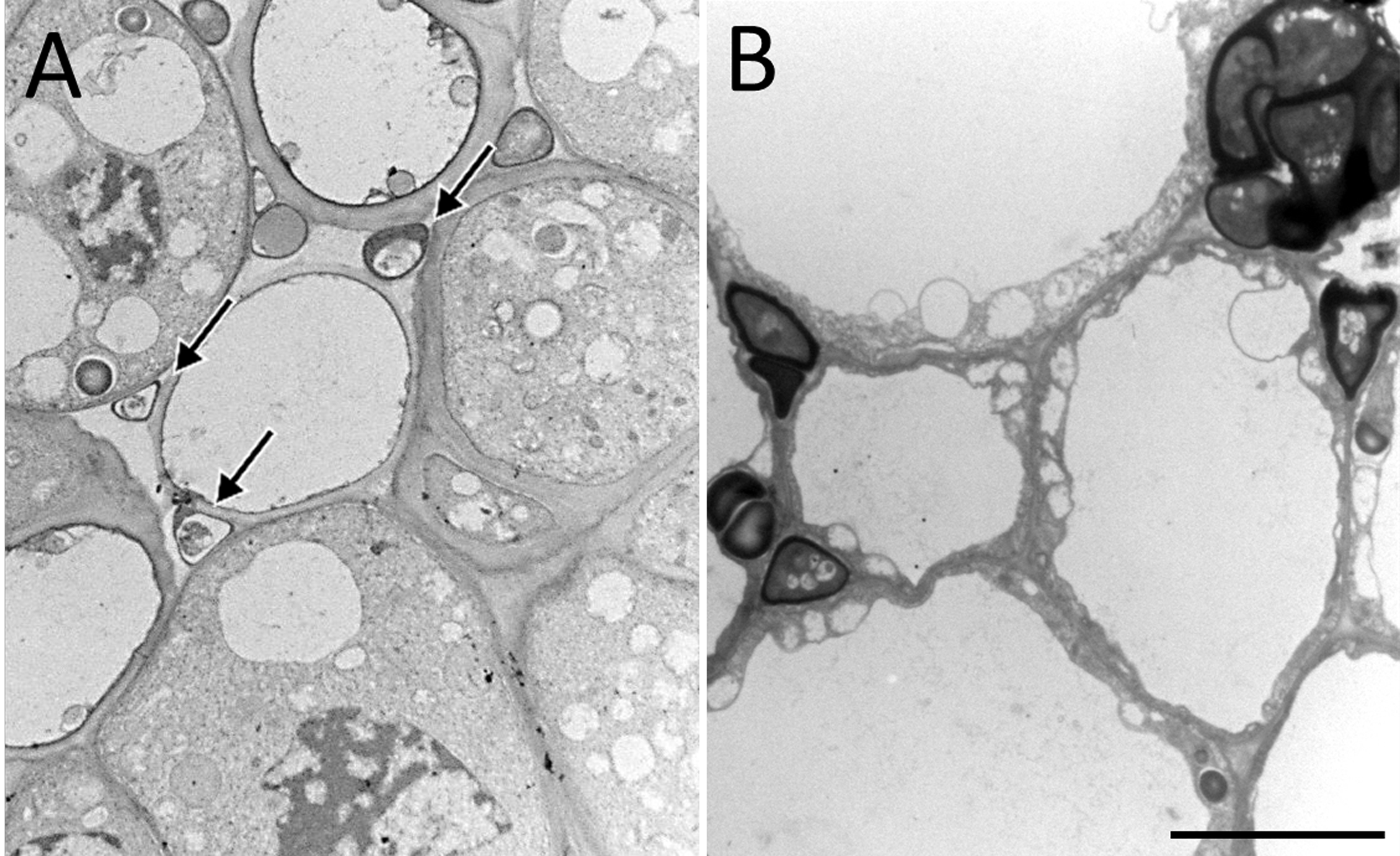

Supplement: S1 Fig — The center of the connective contains more dead fungal cells than does the connective near the pollen sac. Black arrows indicate old hyphae with large vacuoles and dead hyphae. (A) Center of the connective, (B) Connective near the pollen sac. Bars = 5 nm. (TIF) [file pone.0182686.s001.tif]

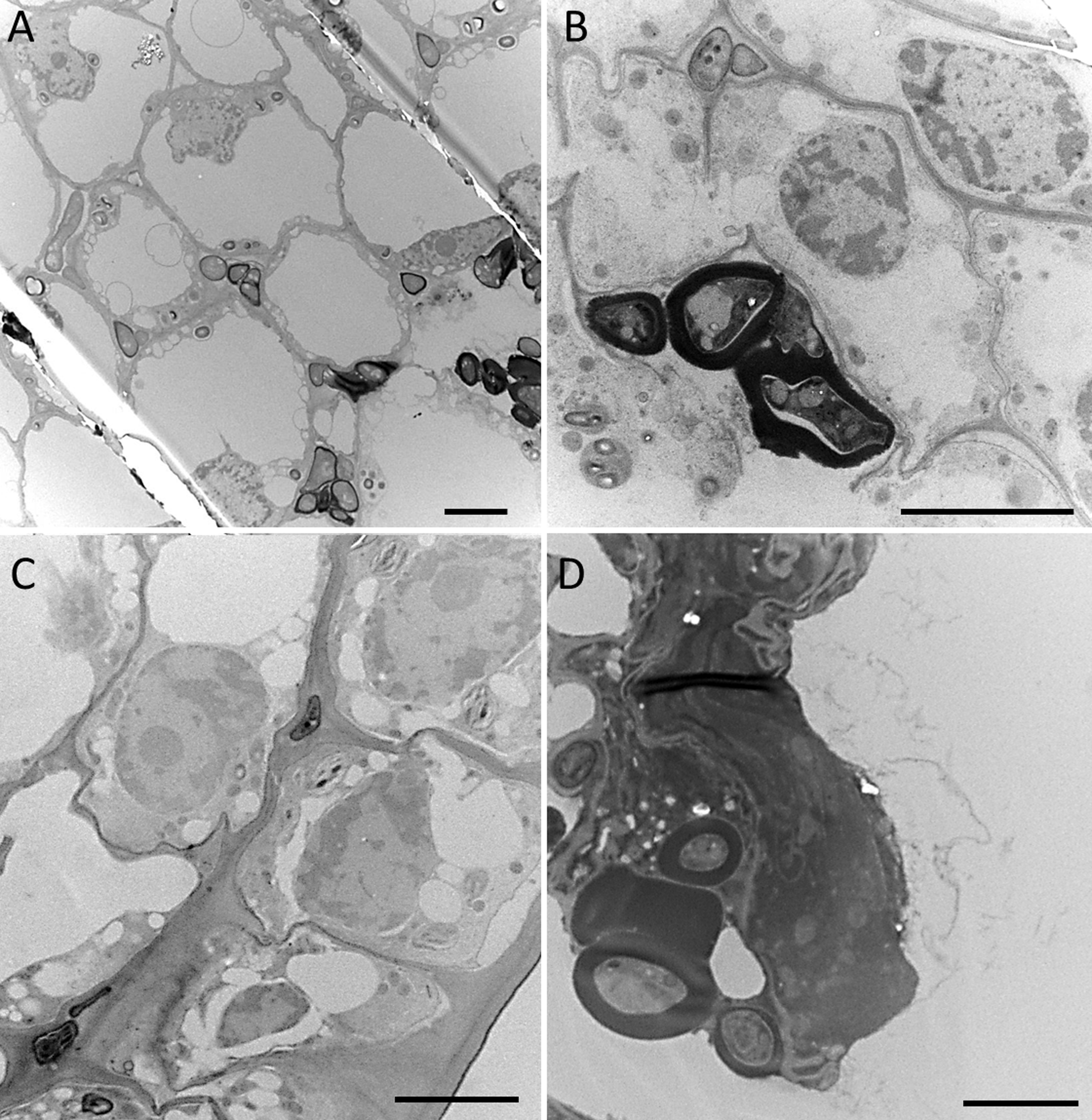

Supplement: S2 Fig — We observed pollen sacs in infected males using serial sections. (A) TEM images using three-dimensional reconstructed images between the endothelium and connective, (B) TEM images using three-dimensional reconstructed images between the tapetum and the middle layer, (C) TEM images using three-dimensional reconstructed images between the epidermis and endothelium, (D) TEM images using three-dimensional reconstructed images in the center of the pollen sac. Bar = 5 μm. (TIF) [file pone.0182686.s002.tif]

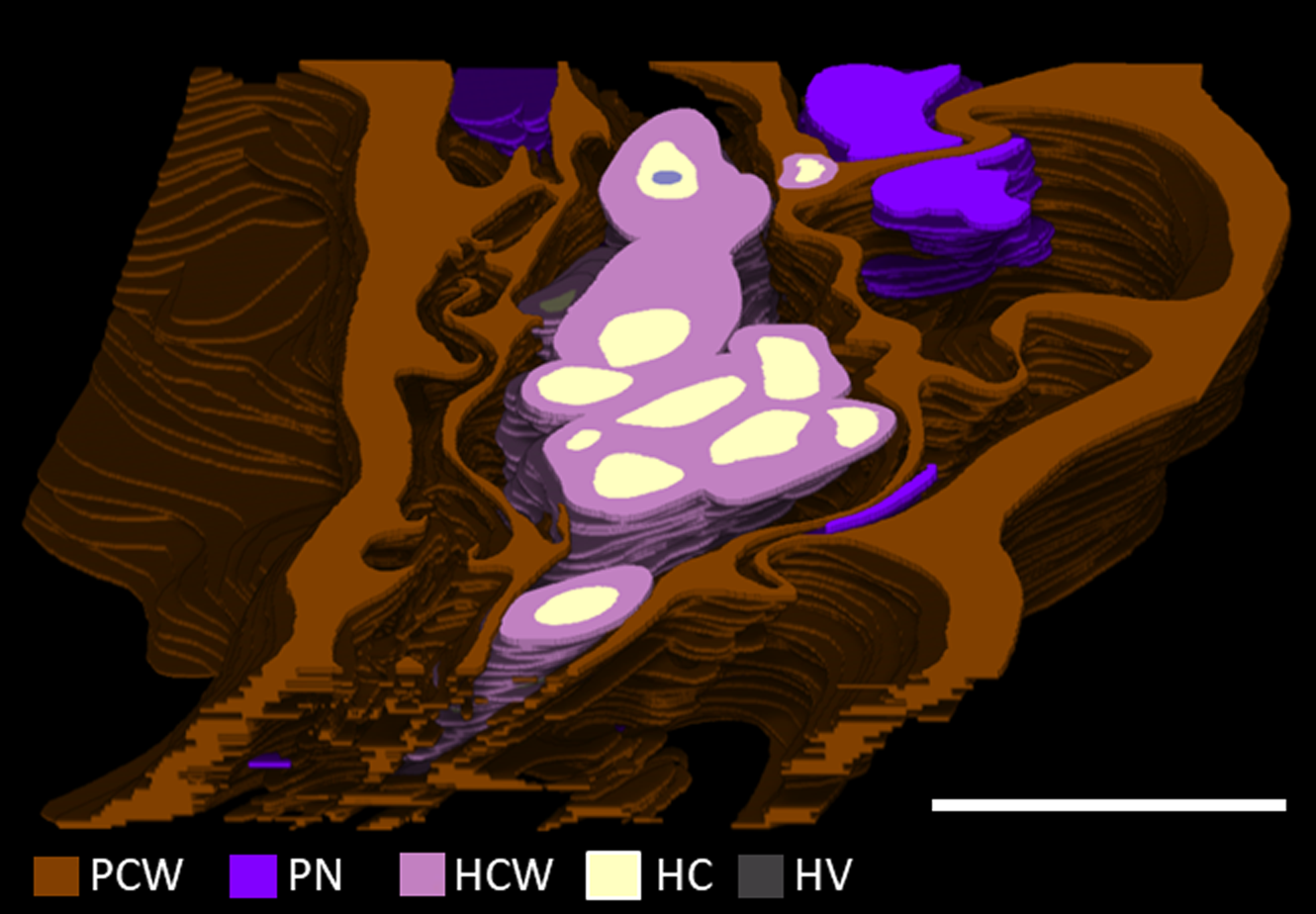

Supplement: S3 Fig — We observed between the epidermis and endothelium in infected male using the serial-section method. PCW: plant cell wall, PV: plant vacuolar, PN: plant nucleus, HCW: hyphae cell wall, HC: hyphae cytoplasm, HV: hyphae vacuolar. Bar = 10 μm. (TIF) [file pone.0182686.s003.tif]
